# Effect of Organic Modifier Types on the Physical–Mechanical Properties and Overall Migration of Post-Consumer Polypropylene/Clay Nanocomposites for Food Packaging

**DOI:** 10.3390/polym13091502

**Published:** 2021-05-07

**Authors:** Eliezer Velásquez, Sebastián Espinoza, Ximena Valenzuela, Luan Garrido, María José Galotto, Abel Guarda, Carol López de Dicastillo

**Affiliations:** 1 Packaging Innovation Center (LABEN-Chile), University of Santiago of Chile (USACH), Obispo Umaña 050, Santiago 9170201, Chile; eliezer.velasquez@usach.cl (E.V.); sebastian.espinoza@usach.cl (S.E.); ximena.valenzuela@usach.cl (X.V.); luan.garrido@usach.cl (L.G.); maria.galotto@usach.cl (M.J.G.); abel.guarda@usach.cl (A.G.); 2 Center for the Development of Nanoscience and Nanotechnology (CEDENNA), University of Santiago of Chile (USACH), Obispo Umaña 050, Santiago 9170201, Chile; 3 Food Science and Technology Department, Technological Faculty, University of Santiago of Chile (USACH), Obispo Umaña 050, Santiago 9170201, Chile

**Keywords:** post-consumer polypropylene, clay, organic modifier, nanocomposite, migration

## Abstract

The deterioration of the physical–mechanical properties and loss of the chemical safety of plastics after consumption are topics of concern for food packaging applications. Incorporating nanoclays is an alternative to improve the performance of recycled plastics. However, properties and overall migration from polymer/clay nanocomposites to food require to be evaluated case-by-case. This work aimed to investigate the effect of organic modifier types of clays on the structural, thermal and mechanical properties and the overall migration of nanocomposites based on 50/50 virgin and recycled post-consumer polypropylene blend (VPP/RPP) and organoclays for food packaging applications. The clay with the most hydrophobic organic modifier caused higher thermal stability of the nanocomposites and greater intercalation of polypropylene between clay mineral layers but increased the overall migration to a fatty food simulant. This migration value was higher from the 50/50 VPP/RPP film than from VPP. Nonetheless, clays reduced the migration and even more when the clay had greater hydrophilicity because of lower interactions between the nanocomposite and the fatty simulant. Conversely, nanocomposites and VPP/RPP control films exhibited low migration values in the acid and non-acid food simulants. Regarding tensile parameters, elongation at break values of PP film significantly increased with RPP addition, but the incorporation of organoclays reduced its ductility to values closer to the VPP.

## 1. Introduction

Polypropylene (PP) is a thermoplastic polymer commonly used to manufacture kitchen utensils, automobile parts, textiles, pipes and packaging. PP was the most demanded plastic by European converters in 2018, representing 19% of the total produced resins (51.2 million tons) [1]. During the last decades, recycling and valorization of post-consumer polypropylene (RPP) have been a global concern to reduce its accumulation in natural environments. Although PP is recyclable, its properties are highly affected due to the mechanical stresses, thermal cycles and degradation reactions during its reprocessing [2]. In an attempt to improve the properties of RPP, nanoparticles, such as carbon nanotubes, zinc oxide and nanoclays, among others, have been incorporated [3].

The use of nanoparticles leads to a high polymer/nanofiller interphase area allowing an adequate energy transfer through the plastic matrix that undergoes stress. Nanoclays are specially used due to their low cost and commercial availability. They are laminar aluminosilicates that can enhance the stiffness and strength of the plastics through the intercalation of the polymer chains between clay galleries [4]. Most of the studies on PP/clay nanocomposites have used virgin resin, and only a few works have focused on the reinforcement of RPP where the nanofillers can potentially act as performance improvers of the degraded plastic, which has a molar mass distribution different than VPP, depending on the grade and source of the recycled polymer [5,6,7,8]. However, these scarce studies have only focused on the morphology and some rheological–mechanical aspects and not on the chemical safety and overall migration of the nanocomposites, which is essential for food packaging materials to guarantee human health and consumer acceptability. Advances in this research line are highly required because: (i) little information on the safety of nanocomposite materials is available; (ii) the variation in the RPP properties and the interactions between components of the nanocomposite and foodstuff, as a function of the type of polymer and organic modifier of the nanoclay, need to be addressed; (iii) the growing new developments of nanotechnology-based materials for food packaging [9,10,11,12].

The bentonite clay is classified under the European Union Regulation 10/2011 (N° FCM 393) as a substance without restriction for food packaging. However, clay minerals need to be organically modified in order to increase their compatibility with hydrophobic polymers as PP. In this regard, a report about the conditions to use ethylene-based polymer/organically modified clay nanocomposites as food contact material from EFSA (European Food Safety Authority) has ruled that a case-by-case safety study is required for each nanocomposite as a function of the type of clay and organic modifier [13]. Therefore, the migration phenomena of substances from polymer/clay nanocomposites to food are still controversial and depend on the type of polymer matrix and food simulant, type and concentration of clay and test conditions [14].

Recently, release studies mainly focused on the migration of Si and Al from polymer/clay nanocomposites based on virgin LDPE, PET and PP have been reported [15,16,17,18,19,20]. Still, safety studies of nanocomposites based on post-consumer plastics have been scarcely carried out [21]. In addition, migration tests become more relevant for recycled plastics due to the high concentration of low molar mass species or oligomers resulting from recycling, which increase substance release [9]. According to our review, no studies have been published about overall migration from RPP and organically modified clay-nanocomposites for food packaging applications. Therefore, in this work, the effect of organic modifier types of a nanoclay (nanoclay hydrophilicity) on the structure, crystallinity, thermal parameters, mechanical properties and overall migration of RPP-based nanocomposites films to different food simulants was analyzed.

## 2. Materials and Methods

### 2.1. Materials

Homopolymer grade virgin polypropylene pellets (VPP) was supplied by Petroquim, S.A (Santiago, Chile) (density: 0.0905 ± 0.005 g mL^−1^). Recycled post-consumer polypropylene pellets (RPPs) were provided by Comberplast, S.A (San Bernardo, Chile). Two organically modified mineral clays (OCs) with different surfactants were used: (i) Non-polar-modified organoclay, OCN, (bulk density: 200–500 kg m^−3^) is a montmorillonite clay modified with a quaternary ammonium salt of tert-alkyl amine; (ii) OCP (bulk density: 300 kg m^−3^) is a montmorillonite with an organic modifier containing polar groups (alkyl-T-bis-2-hydroxyalkyl quaternary ammonium).

### 2.2. Preparation of RPP-Based Nanocomposites

Clay/polymer nanocomposites films based on 50/50 VPP/RPP blend (NC) were obtained because concentrations above 50 wt % of RPP caused simultaneous and significant deterioration of all mechanical parameters (see supplementary file Table S1). Two types of clays (OCN and OCP) were incorporated at concentrations of 1, 3 and 5 wt % with respect to the total mass of nanocomposite, resulting in six different nanocomposites: NC-1OCN, NC-3OCN, NC-5OCN, NC-1OCP, NC-3OCP and NC-5OCP. 

Films were obtained by cast-extrusion in a twin-screw extruder Labtech Scientific LTE-20–40 (Samutprakarn, Thailand), with a temperature profile of 180–190 °C, a screw speed of 20 rpm and 40% torque. The VPP and RPP pellets were previously dried at 100 °C, and the clays were dried at 40 °C in a vacuum oven, both for 48 h. Blends were homogenized before adding to the extruder hopper. Additionally, VPP, RPP and 50/50 VPP/RPP control films were prepared under the same conditions.

### 2.3. Characterization of the Films

#### 2.3.1. Structural Analysis

The clays and nanocomposites were characterized by X-ray diffraction (XRD) in order to determine the interlaminar distance in the clays and the type of structure of the nanocomposites. The equipment used was a Siemens D5000 brand X-ray diffractometer (30 mA, 40 KV, München, Germany). The samples were explored in reflection mode using an incident Cu-Kα radiation and a step of 0.02° min^−1^, in a range 2Ɵ = 2–9°. The interlaminar distance (d_001_) was determined using Bragg’s Law following Equation (1):

(1) $$\mathrm{sen}\left(\theta \right)=\frac{n\lambda }{2d}$$

where d is the interlaminar distance in the clay, θ is the angle between the incident rays and the scattering plane, n is equal to 1 and λ is the wavelength of the X-ray (0.154 nm).

#### 2.3.2. Thermal Analysis

The thermal stability of the samples (6–8 mg) was determined in a Mettler Toledo Gas Controller GC20 Stare System TGA/DSC equipment using a heating program from 30 to 600 °C with a heating rate of 10 °C min^−1^ under nitrogen atmosphere. The decomposition initiation temperature at 5% mass loss (T_onset_), the temperature at the maximum degradation rate (T_d_) and the mass loss at T_d_ were determined.

DSC analysis was carried out in a calorimeter Mettler-Toledo model STAR 822e (Greinfensee, Switzerland) coupled to a cooling unit (HAAKE, USA). The samples in aluminum capsules (6–8 mg) were subjected to two thermal scans, heating from 25 to 250 °C and cooling from 250 to 25 °C, at a constant speed of 10 °C min^−1^ under nitrogen atmosphere. The melting (T_m_) and crystallization temperatures (T_c_), melting (ΔH_m_) and crystallization enthalpies (ΔH_c_) and the percent crystalline fraction were registered. The crystallinity was calculated from Equation (2), relating the PP melting heat (ΔH_m_) of the sample and the theoretical melting heat of 100% crystalline polypropylene (ΔH_100_):

(2) $$X_{c}=\frac{\Delta H_{m}}{\Delta H_{100}\times X_{\mathrm{PP}}}*100\left(\%\right)$$

where X_c_ is the percentage of crystalline fraction (%), ΔH_m_ is the melting enthalpy of the sample (J g^−1^), ΔH_100_ is the melting enthalpy of fully crystalline polypropylene equal to 206.7 J g^−1^ [22] and X_PP_ is the mass fraction of polypropylene in the film (%). The results of thermal parameters and crystallinity were reported as the mean and standard deviation of two measurements.

#### 2.3.3. Mechanical Properties

Tensile parameters of the films such as Young’s modulus (YM), tensile strength (TS) and elongation at break (EB) were determined in a Zwick Roell universal machine (model BDO-FB 0.5 TH, Ulm, Germany) according to the ASTM D882 normative. Specimens with dimensions 16.5 cm × 2.4 cm were cut and conditioned at 23 °C for 48 h in a desiccator with RH of 50% ± 10%. The separation distance between the jaws was 50 mm and the crosshead speed was 500 mm min^−1^. The results were reported as the mean and standard deviation of 15 measurements.

#### 2.3.4. Overall Migration Analysis

Overall migration tests were conducted according to EU Regulation N° 10/2011 and UNE-EN 1186 normative. The nanocomposites that reached the best tensile performance, VPP, RPP and VPP/RPP 50/50 control films, were analyzed. Double-sided, total immersion migration tests were conducted using an area-to-volume of food simulant ratio of 6 dm^2^ L^−1^. Used as acidic, non-acid hydrophilic and fatty food simulants were 3% acetic acid, 10% ethanol solutions and isooctane, respectively. Tests were conducted at 40 °C for 10 days in the acid and non-acid simulant, and at 20 °C for 48 h in the substitute fatty food simulant according to the substitute conventional conditions for isooctane of UNE EN 1186 normative.

After the contact time, each food simulant was transferred to dry crucibles that were previously weighed. Food simulants were evaporated until reaching constant mass and the overall mass that migrated per dm^2^ was quantified by gravimetry. The results were reported as the mean and standard deviation of four measurements.

### 2.4. Statistical Analysis

Data were analyzed through an analysis of variance (ANOVA) and Fisher’s multiple range test using the Statgraphic plus 5.1 program. The experimental design was the random type where a *p*-value less than 0.05 indicated significant differences in the measurements between samples and were highlighted with different superscript letters.

## 3. Results

### 3.1. Structural Properties

**Figure 1:**
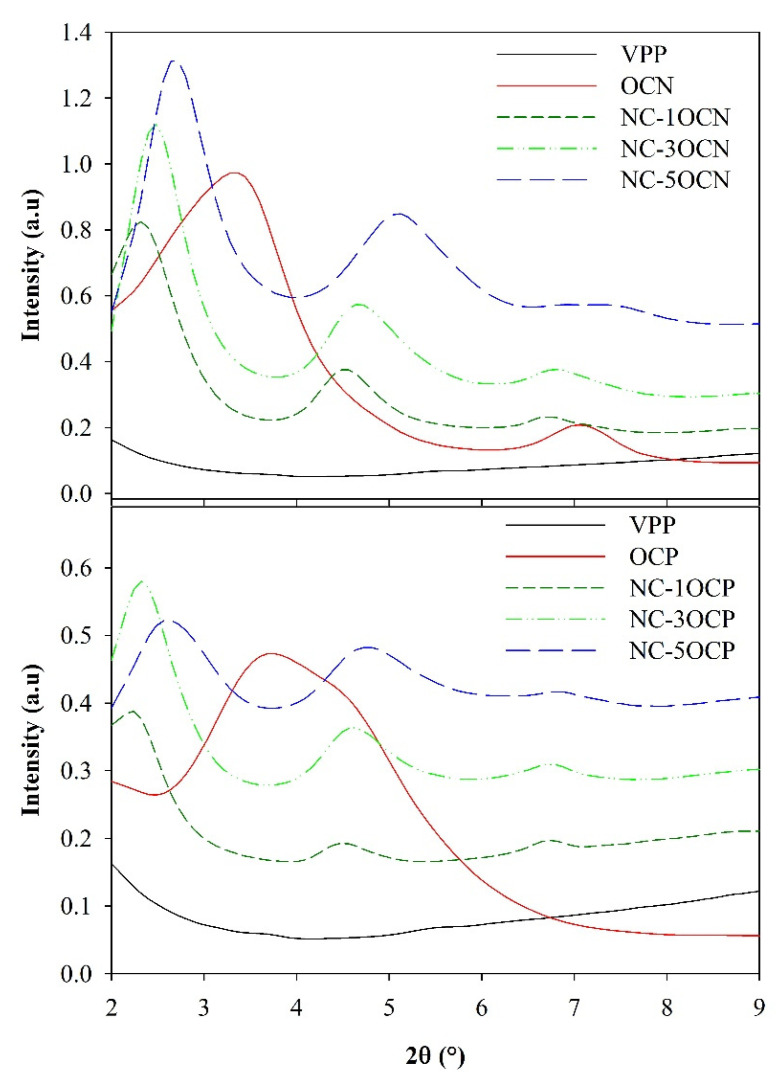
XRD diffractograms of organoclays OCN and OCP, and their corresponding nanocomposites.

Nanocomposites presented a combination of intercalated and tactoid structures except nanocomposites containing OCN clay at 1 and 3 wt %, whose XRD diffractograms evidenced only intercalated structures indicating better nanoclay dispersion. Despite differences in polarity between PP and clays, intercalated structures were obtained by high shear and dispersive forces during extrusion. The highest hydrophobicity of the OCN organic modifier promoted greater compatibility with PP chains and their better intercalation between clay mineral layers. As is shown in Table 1 and Figure 1, OCN pure clay presented two peaks at 3.32° and 7.03° (peaks a and b) while nanocomposites with 1 and 3 wt % OCN displayed three peaks (c, d and e), all of them associated with intercalated structures and increments of interlaminar distance. In contrast, tactoid structures or agglomerates were formed at 5 wt % of OCN evidenced by a reduction of the interlayer space (peak e) (Table 1). 

The reduction of the interlaminar distance can be attributed to partial decomposition of the organic modifier confined between clay mineral layers, as it has been reported for clay/polymer nanocomposites [21,23]. This phenomenon exhibited a more significant effect on nanocomposites with OCP evidenced through a greater reduction of the interlayer space regardless of clay concentration, from 24 to 12.9–19.7 Å (peaks d and e, Table 1). Likewise, the chain intercalation was diminished and lower interlaminar distances were obtained when clay concentration in the nanocomposites increased (Table 1).

On the other hand, the diffractogram of pure OCP presented the characteristic peak of this clay at 3.68° (Figure 1). Meanwhile, nanocomposites NC-OCP presented three peaks, the one at lower angles associated with an increase of the interlayer distance (peak c) and the other two peaks related to the reduction of the interlaminar distance (peaks d and e), resulting in combined intercalated/tactoid structures (Table 1). 

### 3.2. Thermal Properties

#### 3.2.1. Differential Scanning Calorimetry 

During the first heating process, VPP film presented a unique endothermic transition at 164.5 °C, corresponding to the melting point of this polymer (Table 2). RPP-based films showed another endothermic transition at 124 °C, which indicated the presence of traces of high-density polyethylene (HDPE), low-density linear polyethylene (LLDPE) or their blends due to their close melting transitions, as principal pollutants of the RPP. These two transitions were also found separately by Vynche et al. (2020) for recycled polypropylene from electronic equipment with known HDPE composition (10 wt %) [24]. Traces of several polymers can remain commonly mixed with post-consumer PP after collection and separation during the recycling process, especially LLDPE and HPDE, because of the similarity between their specific gravity and combined uses in the same article [25]. For example, recycled plastic caps are mainly composed of PP, but LLDPE and HDPE are used to manufacture part of seal caps, plugs and flip caps.

Besides, the melting peak of PP can be associated with homopolymer or copolymer polypropylene in the post-consumer material, considering both polymer grades have similar melting behavior [24]. Regarding the melting transition of nanocomposites, the thermograms manifested two shoulders at 164 and 167 °C approx., associated with the formation of different crystalline structures, excluding nanocomposites at 1 wt % OCN. 

All films had a highly ordered structure with similar polypropylene crystallinities of 49% but decreased to 43% for the pure RPP film. This fact can be attributed to a hindrance of PP chain packing caused by a greater portion of pollutant polymers with different chemical structures in the RPP sample. Specifically, polyethylene, a common pollutant of RPP, whose highest concentration in the RPP film was demonstrated through the highest heat (ΔH_m1_) required for its melting (Table 2). In the case of linear HDPE, its higher mobility can inhibit PP crystallization before phase separation, similarly as reported for virgin PP/HDPE blends for electrical and electronic applications [24]. Besides, similar crystallinities for nanocomposites revealed the absence of a nucleating effect of the clays on VPP/RPP blend and only promoted different crystal structures, especially at high clay concentration.

During the cooling process, VPP film showed a single exothermic transition at 113.6 °C corresponding to the crystallization from the molten polymer (Table 2). Meanwhile, RPP-based films presented two transitions related to PP and PE crystallizations at 123 and 105 °C, respectively, confirming the presence of LLDPE and/or HDPE. The crystallization temperature found in the recycled material around 105 °C was between the values reported for virgin LLDPE and HPDE [25]. Likewise, the crystallization temperature displacement to higher values, from 113 °C for VPP to 123 °C for RPP-based films, can be explained through the acceleration of the crystal formation by the nucleation action of the recycled polymer chains with lower molar mass than VPP. The molar mass reduction of the PP and other polyolefins due to thermal–mechanical degradation during reprocessing has been previously reported [26,27]. Likewise, a higher enthalpy (ΔH_c1_) of RPP film indicated a higher PE proportion in this sample, as was expected. 

The slight differences between enthalpies of the thermal transitions in the nanocomposites can be associated with heterogeneities of the recycled polymer batch in regards to pollutant composition and molar mass distribution.

#### 3.2.2. Thermogravimetric Analysis

**Figure 2:**
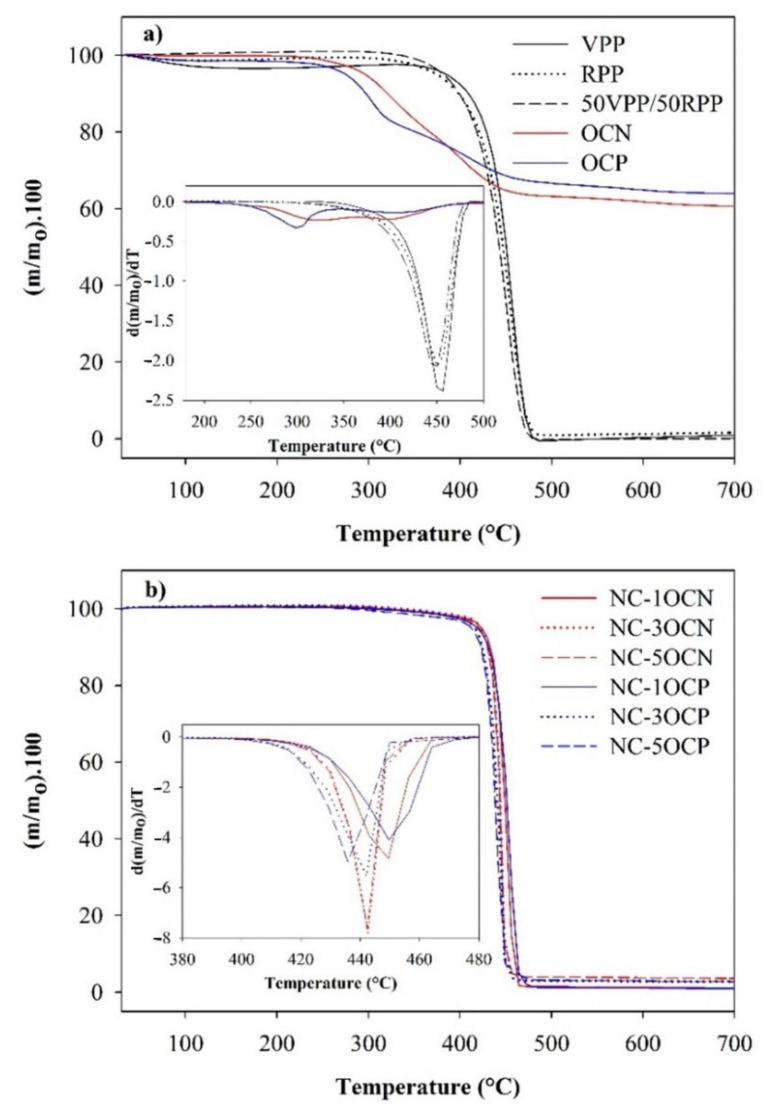
TGA and DTG of (**a**) organoclays and control films and (**b**) nanocomposites.

TGA and DTG curves are shown in Figure 2. The onset decomposition temperatures of the RPP-based films without nanoclay were lower than the temperature values of the VPP film (Table 3). The same tendency was observed for maximum degradation rate temperatures up to a lesser extent. This fact is in concordance with the greater susceptibility of the degraded shorter chains in the RPP to thermal decomposition. However, TGA parameters of the blend with 50 wt % of RPP remained close to the parameters of the VPP film.

On the other hand, the incorporation of nanoclays into the VPP/RPP blend improved its thermal stability shifting onset decomposition at higher T_onset_ values, with a less significant effect on nanocomposites with OCP. These results agreed with (i) the lower T_onset_ for pure OCP (267.2 °C) compared to OCN (296.4 °C), indicating that earlier degradation of the organic modifier of OCP accelerated the decomposition of the nanocomposite, and (ii) the organic surfactant of pure OCP degraded with a maximum rate at a lower temperature (299 °C) than OCN (327 °C) (Table 3). Both organic modifiers degraded in two stages according to the confinement of the surfactant between clay mineral layers, as is shown in Figure 2. These phenomena are observed during TGA analysis of the films and clays but it is worth mentioning that extrusion temperature for obtaining films (180–190 °C) was far below T_onset_ of the pure clays. 

In addition, regardless of the type of clay, the decomposition tended to start at lower T_onset_ values when clay concentration was increased. Meanwhile, T_d_ remained practically unchangeable in the nanocomposites compared to the control VPP/RPP blend when 1 wt % of the nanoclay was incorporated but decreased at higher concentrations (3 and 5 wt %). This fact can be attributed to a higher amount of the nanofiller poorly attached to the PP matrix at a higher concentration of clay, which tended to decompose, although less significantly for OCN with a more hydrophobic organic modifier. Nonetheless, regardless of the type of clay, all nanocomposites had mass losses lower than the VPP/RPP control film at the maximum degradation rate.

### 3.3. Mechanical Properties 

The tensile parameters of the 50VPP/50RPP blend were between values of the VPP and RPP films. Increasing RPP concentration deteriorated the mechanical performance of the films, as was expected (Table 4). The YM and the TS of the films decreased with RPP incorporation possible due to several factors: (i) RPP was composed of short chains with lower molar masses compared to VPP chains, reducing stiffness and TS because oligomers did not support stress forces during traction [3], and (ii) the presence of PE and other possible pollutants in the RPP film, such as copolymer grade polypropylene containing ethylene, could have affected the stress transfer based on miscibility differences between polymers [24,28]. Likewise, according to the thermal analysis, the lowest rigidity of the RPP film can also be associated with its lowest crystallinity (see Table 2). 

Deformation at break values drastically increased as a function of RPP concentration in films without clay, showing a 2.8 and 9.6-fold increase with respect to virgin material for VPP/RPP 50/50 blend and 100% RPP sample, respectively. This fact can be associated with the presence of ductile polymers such as polyethylene in the RPP. For example, EB values above 800% have been reported for several LLDPE and HDPE grades [25,29,30].

On the other hand, mechanical parameters of the nanocomposites depended more on the resulting polymer/clay structures formed than their crystallinities, which were similar at all concentrations (see Table 4). YM tended to increase at 1 wt % nanoclay compared to the control VPP/RPP blend associated with the rigidity of the silicate structures, which display a high aspect ratio, and the immobilization of polymer chains intercalated between the clay mineral layers. Nonetheless, the elastic modulus of the nanocomposites containing OCP at 3 and 5 wt % decreased with a statistically significant difference, which can be attributed to the lower intercalation of PP chains between OCP layers at high clay concentration, as was observed earlier by XRD analysis. Furthermore, TS decreased at high clay concentration, possibly due to the greater formation of tactoid structures and an apparent poorer adhesion clay/polymer matrix observed by XRD and TGA, respectively. 

Deformation at break values of the nanocomposites were significantly lower than EB values of VPP/RPP blend and closer to the VPP deformation (Table 4). These results were statistically similar regardless of the clay type and concentration. Nonetheless, EB of the nanocomposites with OCN were significantly lower than in the VPP. Besides, EB tended to slightly decrease with clay concentration, possibly due to a higher amount of tactoids that hindered energy dissipation through nanocomposite when this undergoes tensile stress causing the premature breakage [23]. Thus, interestingly, the incorporation of nanoclays not only counteract the effect of the degraded PP chains but also the great impact of the presence of pollutant polymers on the deformation behavior of the RPP. Higher standard deviations for EB results can be associated with different chain length distributions and pollutant compositions for each polymer batch, creating different numbers of tension points in the film.

Based on these results, the NC-1OCN nanocomposite had the most improved mechanical parameters, mainly elastic modulus and tensile strength, in accordance with the higher intercalation of PP chains between clay mineral layers, as it was previously observed through XRD due to the greater affinity of the hydrophobic organic modifier of OCN with PP.

### 3.4. Overall Migration Results

If recycled polymers are intended to be applied as food contact packaging materials, analyzing the effect of the recycling processes on their safety is necessary. Thus, overall migration studies of RPP nanocomposites under three different food simulants were carried out to understand their dependence on the type of packaged food. The overall migration values from VPP, RPP, 50VPP/50RPP and NC films at 1 wt % of clay, which had the best mechanical performance are shown in Table 5. 

The overall migration from 100% VPP and RPP control films was determined in a fatty food simulant, considering that this medium caused the maximum migration due to the greater affinity between fatty simulant and PP. A higher RPP percentage in the films without clay significantly increased migration, with a 2.9 and 4-fold increase for 50% and 100% RPP samples, respectively.

The current European Union Regulation (No. 10/2011 of the Commission of 14 January 2011) establishes an overall migration limit of 10 mg dm^−2^ from plastics to be used in direct contact with food, equivalent to 60 mg per kg of food occupying a volume of a cubic package with 6 dm^2^ of total area. Thus, RPP-based films in the fatty simulant at the conditions studied exceeded this limit. However, the EU normative (N° 10/2011 with modification 2016/1416) considers applying fat reduction factors to migration values for food categories using a D2 fat simulant at the predictable conditions of use.

In the fatty simulant, the incorporation of the nanoclays in the PP matrix decreased the overall migration probably due to tortuous pathways created by clay mineral layers that increased the barrier properties, and therefore, hindered migration from the polymer. This phenomenon has been similarly observed for RPET/clay nanocomposites [21]. 

The nanocomposite containing OCP exhibited a more significant decrease of the overall migration in the fatty simulant, probably due to some polarity of the OCP organic modifier containing hydroxyl groups, which inhibited the interaction of the hydrophobic simulant with the nanocomposite.

Conversely, overall migrations from 50VPP/50RPP control and nanocomposites films in the acidic and non-acidic aqueous simulants were far below (<1 mg dm^−2^) than migration values obtained in fatty simulants. Besides, no statistically significant differences were observed between the 50VPP/50RPP control film and nanocomposites. However, it can be noted that nanocomposites with OCP showed a slight tendency to increase migration compared to films with OCN. These results highlighted the effect of the higher hydrophilicity of the OCP, which increased the affinity between the nanocomposite and aqueous simulants, causing a greater penetration of the simulant in the polymeric matrix and a higher release of their components.

## 4. Conclusions

The nanocomposite at 1 wt % of OCN had the best thermal and mechanical performance at the studied conditions. The highest intercalation grade of PP between clay mineral layers was reached with OCN because its organic modifier is more hydrophobic. Pollutant polymer traces in the RPP drastically increased its ductility. Interestingly, nanoclay incorporation caused a reduction of the elongation at break of the RPP/VPP film, although it did not significantly increase Young’s modulus and tensile strength, which were instead reduced at high clay percentage. The latter can be due to a greater amount of tactoids or clay particles poorly attached to the polymer matrix, considering crystallinities were similar for all nanocomposites.

Conversely, the overall migration values of nanocomposites to 3% acetic acid and 10% ethanol food simulants were lower than 1 mg dm^−2^, far below the limit ruled by current European legislation. Meanwhile, the overall migration of free-clay RPP-based films and the nanocomposites in fatty simulant exceeded that limit, and, although the incorporation of nanoclays exerted a barrier effect, migration values were still higher than the allowed limit. Nonetheless, fat reduction factors can be applied in an adequate fatty simulant according to current European legislation for plastics to be used in specific food categories and predictable use conditions. Finally, it is highlighted that the clay with the most hydrophobic organic modifier improved the thermal properties, the intercalation grade and some mechanical parameters of the VPP/RPP blend, but increased the affinity of the fatty simulant towards nanocomposites and overall migration to that food simulant.

## Figures and Tables

**Table 1:** The 2θ angles and interlaminar distances in the pure clays and nanocomposites.

Sample	2θ				d (Å)			
**Clays**	**a**		**b**		**a**		**b**	
OCN	3.32		7.03		26.6		12.6	
OCP	3.68		-		24.0		-	
**Film**	**c**	**d**		**e**	**c**	**d**		**e**
NC-1OCN	2.32	4.52		6.69	38.0	19.5		13.2
NC-3OCN	2.49	4.67		6.74	35.4	18.9		13.1
NC-5OCN	2.69	5.08		7.53	32.8	17.4		11.7
NC-1OCP	2.25	4.47		6.74	39.2	19.7		13.1
NC-3OCP	2.35	4.59		6.76	37.5	19.2		13.1
NC-5OCP	2.61	4.78		6.82	33.8	18.5		12.9

**Table 2:** DSC parameters and crystallinities of the PP films.

**Films**	**First Heating Process**							
	**T_m1_ (°C)**	**ΔH_m1_ (J g^−1^)**		**T_m2_ (°C)**	**T_m3_ (°C)**	**ΔH_m2_ (J g^−1^)**		**X_c_ (%)**
VPP	-	-		164.5 ± 1.3 ^ab^	-	102.1 ± 7.1 ^c^		49.4 ± 3.4 ^b^
RPP	124.0 ± 0.3 ^a^	2.18 ± 0.05^c^		165.4 ± 1.7 ^b^	-	89.6 ± 0.1 ^a^		43.3 ± 0.1 ^a^
50VPP/50RPP	123.6 ± 0.7 ^a^	0.63 ± 0.16 ^b^		165.6 ± 0.7 ^b^	-	101.6 ± 1.6 ^c^		49.2 ± 0.8 ^b^
NC-1OCN	123.9 ± 0.1 ^a^	0.63 ± 0.06 ^b^		165.6 ± 0.4 ^b^	-	98.5 ± 1.2 ^bc^		48.1 ± 0.6 ^b^
NC-3OCN	124.0 ± 0.2 ^a^	0.60 ± 0.10 ^ab^		164.6 ± 1.2 ^ab^	167.6 ± 0.6 ^a^	97.7 ± 5.5 ^bc^		48.7 ± 2.7 ^b^
NC-5OCN	124.5 ± 0.8 ^a^	0.52 ± 0.19 ^ab^		163.3 ± 0.2 ^a^	167.2 ± 0.8 ^a^	96.9 ± 1.6 ^abc^		49.3 ± 0.8 ^b^
NC-1OCP	123.7 ± 0.4 ^a^	0.48 ± 0.04 ^ab^		163.8 ± 0.6 ^ab^	166.9 ± 0.6 ^a^	100.6 ± 0.7 ^c^		49.2 ± 0.3 ^b^
NC-3OCP	124.7 ± 0.1 ^a^	0.30 ± 0.04 ^a^		163.9 ± 0.1 ^ab^	167.6 ± 0.9 ^a^	95.2 ± 2.5 ^abc^		47.5 ± 1.2 ^b^
NC-5OCP	124.4 ± 0.5 ^a^	0.58 ± 0.18 ^b^		164.7 ± 0.1 ^ab^	167.6 ± 0.4 ^a^	91.6 ± 1.6 ^ab^		46.7 ± 0.8 ^ab^
**Films**	**Cooling Process**							
	**T_c1_ (°C)**		**ΔH_c1_ (J g^−1^)**		**T_c2_ (°C)**		**ΔH_c2_ (J g^−1^)**	
VPP	-		-		113.6 ± 0.4 ^a^		129.9 ± 10.5 ^c^	
RPP	105.6 ± 0.1 ^a^		1.94 ± 0.01 ^c^		123.4 ± 0.1 ^bc^		110.9 ± 1.2 ^a^	
50VPP/50RPP	105.8 ± 0.1 ^a^		0.41 ± 0.11 ^a^		123.7 ± 0.3 ^c^		122.6 ± 4.6 ^abc^	
NC-1OCN	105.6 ± 0.2 ^a^		0.33 ± 0.03 ^a^		123.1 ± 0.2 ^bc^		117.0 ± 2.1 ^ab^	
NC-3OCN	107.3 ± 2.4 ^a^		-		123.4 ± 0.4 ^bc^		114.1 ± 9.4 ^ab^	
NC-5OCN	105.4 ± 0.1 ^a^		0.32 ± 0.04 ^a^		122.9 ± 0.4 ^b^		117.6 ± 1.8 ^abc^	
NC-1OCP	105.4 ± 0.1 ^a^		0.56 ± 0.02 ^b^		123.2 ± 0.0 ^bc^		125.7 ± 2.5 ^bc^	
NC-3OCP	105.6 ± 0.2 ^a^		0.33 ± 0.02 ^a^		123.0 ± 0.2 ^b^		114.4 ± 3.7 ^ab^	
NC-5OCP	105.6 ± 0.2 ^a^		0.40 ± 0.01 ^a^		123.0 ± 0.1 ^b^		111.0 ± 4.2 ^a^	

X_c_ corresponds to PP crystallinity. Superscripts a–c indicate significant differences for the same parameter among films according to the ANOVA analysis and Fisher LSD test (*p* < 0.05).

**Table 3:** TGA parameters of PP films and organoclays.

Samples	T_onset_ (°C)	T_d_ (°C)	Mass Loss at T_d_ (wt %)
VPP	384.5	457.1	65.6
RPP	374.5	454.8	65.6
50VPP/50RPP	381.8	451.1	66.5
NC-1OCN	423.6	450.1	55.0
NC-3OCN	423.3	444.1	50.9
NC-5OCN	420.6	443.7	50.7
NC-1OCP	418.8	452.7	56.3
NC-3OCP	416.3	441.8	53.3
NC-5OCP	412.0	439.0	52.4
OCN	296.4	327 and 402	27.2
OCP	267.2	299 and 404	27.1

T_onset_ and T_d_ correspond to onset decomposition at 5 wt % mass loss and maximum degradation rate temperatures, respectively.

**Table 4:** Tensile parameters of PP films.

Films	YM (MPa)	TS (MPa)	EB (%)
VPP	533± 145 ^a^	25.5 ± 3.6 ^a^	72 ± 22 ^bc^
RPP	349 ± 71 ^d^	19.9 ± 2.8 ^c^	692 ± 28 ^a^
50VPP/50RPP	473 ± 123 ^ab^	23.5 ± 2.1 ^b^	199 ± 209 ^b^
NC-1OCN	530 ± 75 ^a^	24.0 ± 1.6 ^ab^	36 ± 15 ^d^
NC-3OCN	493 ± 114 ^ab^	19.3 ± 1.7 ^cd^	21 ± 6 ^d^
NC-5OCN	425 ± 96 ^bc^	17.1 ± 1.0 ^e^	24 ± 8 ^d^
NC-1OCP	538± 68 ^a^	22.8 ± 1.6 ^b^	59 ± 42 ^cd^
NC-3OCP	379 ± 96 ^cd^	17.9 ± 2.0 ^de^	56 ± 59 ^cd^
NC-5OCP	334 ± 69 ^d^	12.9 ± 1.1 ^f^	41 ± 18 ^d^

YM: Young’s modulus. TS: tensile strength. EB: elongation at break. Superscripts a–f indicate significant differences for the same parameter among films, according to the ANOVA analysis and Fisher LSD test (*p* < 0.05).

**Table 5:** Overall migration from control and nanoreinforced films to different food simulants.

Food Simulant	Film	Overall Migration (mg dm^−2^)
Fatty	VPP	6.72 ± 0.49 ^a^
	RPP	27.19 ± 0.86 ^e^
	50VPP/50RPP	19.34 ± 0.15 ^d^
	NC-1OCN	17.86 ± 0.14 ^c^
	NC-1OCP	16.62 ± 0.11 ^b^
Non acid	50VPP/50RPP	0.30 ± 0.07 ^a^
	NC-1OCN	0.42 ± 0.38 ^a^
	NC-1OCP	0.66 ± 0.32 ^a^
Acid	50VPP/50RPP	0.37 ± 0.07 ^a^
	NC-1OCN	0.46 ± 0.16 ^a^
	NC-1OCP	0.56 ± 0.04 ^a^

Superscripts a-d indicate significant differences among films for each type of simulant according to the ANOVA analysis and Fisher LSD test (*p* < 0.05).

## Data Availability

Not applicable.

## Supplementary Materials

The following are available online at https://www.mdpi.com/article/10.3390/polym13091502/s1, Table S1: Tensile parameters of VPP/RPP films.
