# Supplementary material for: Effect of Organic Modifier Types on the Physical–Mechanical Properties and Overall Migration of Post-Consumer Polypropylene/Clay Nanocomposites for Food Packaging
Source: Polymers (Basel). 2021 May 7;13(9):1502. doi: 10.3390/polym13091502 (PMC8125780; doi:10.3390/polym13091502)
Supplement: Supplementary file 1 [file polymers-13-01502-s001.zip › polymers-1202366-supplementary.pdf]

polymers

Article

Effect of Organic Modifier Types on the Physical–Mechanical Properties and Overall Migration of Post-Consumer Polypropylene/Clay Nanocomposites for Food Packaging

Eliezer Velásquez [1,2], Sebastián Espinoza [1], Ximena Valenzuela [1,2], Luan Garrido [1], María José Galotto [1,2,3], Abel Guarda [1,2,3] and Carol López de Dicastillo [1,2,3,*]

1 Packaging Innovation Center (LABEN-Chile), University of Santiago of Chile (USACH), Obispo Umaña 050, Santiago 9170201, Chile; eliezer.velasquez@usach.cl (E.V.); sebastian.espinoza@usach.cl (S.E.); ximena.valenzuela@usach.cl (X.V.); luan.garrido@usach.cl (L.G.); maria.galotto@usach.cl (M.J.G.); abel.guarda@usach.cl (A.G.)
2 Center for the Development of Nanoscience and Nanotechnology (CEDENNA), University of Santiago of Chile (USACH), Obispo Umaña 050, Santiago 9170201, Chile
3 Food Science and Technology Department, Technological Faculty, University of Santiago of Chile (USACH), Obispo Umaña 050, Santiago 9170201, Chile
* Correspondence: analopez.dediscastillo@usach.cl; Tel.: +56-951377492

S-1. Mechanical Properties of VPP/RPP Blends

In a preliminary stage, different VPP/RPP blends (100/0, 75/25, 50/50, 25/75, 0/100) were extruded in order to define the concentration of RPP that produced a deterioration of tensile mechanical parameters (Young's modulus, tensile strength and elongation at break). VPP and RPP pellets were previously dried at 100°C for 48 h and the films were obtained in a Labtech Scientific LTE-20-40 twin-screw extruder (Thailand), with a temperature profile between 180-190 °C, a screw speed of 20 rpm and 40% torque.

VPP/RPP films were analyzed by tensile tests in a Zwick Roell universal machine (model BDO-FB 0.5 TH, Ulm, Germany) according to ASTM D882 standard to determine Young's modulus (YM), tensile strength (TS) and elongation at break (EB). Samples sizing 16.5 cm x 2.4 cm were analyzed. The specimens were previously conditioned at 23°C for 48 h in a desiccator at 50% ± 10% RH. The separation distance between the grips was 50 mm and the test speed was 500 mm min^{-1}. The results were reported as the mean and standard deviation of 15 measurements.

The films with RPP concentration equal to or higher than 50 wt% evidenced a significant deterioration of all mechanical parameters and high deviations in the elongation at break values (Table S1). Therefore, VPP/RPP 50/50 was selected as the target blend because it presented significant deterioration and its processing through melt-extrusion did not cause great problems during film formation. Subsequently, the control 50VPP/50RPP films were prepared again during the extrusion of developed nanocomposites for comparative purposes in order to consider the standard deviations inherent to the dispersion of commercial pellets and environmental and/or experimental factors during extrusion and tensile testing.

Table S1. Tensile parameters of VPP/RPP films.

Films	YM (MPa)	TS (MPa)	EB (%)
VPP	851 ± 110^{e}	32 ± 3.3^{d}	46 ± 20^{a}
75VPP/25RPP	635 ± 45^{d}	26 ± 1.3^{c}	76 ± 32^{ab}
50VPP/50RPP	541 ± 60^{c}	24 ± 1.7^{b}	155 ±157^{b}
25VPP/75RPP	448 ± 45^{b}	22 ± 1.3^{a}	138 ± 143^{ab}
RPP	361 ± 41^{a}	21 ± 2.4^{a}	594 ± 298^{c}

Superscripts a-e indicate significant differences for the same parameter among films according to ANOVA analysis and Fisher LSD test ($p < 0.05$).
